# Global research trends and hotspots in human immunodeficiency virus-associated cervical cancer (1990–2025): a multi-database bibliometric analysis

**DOI:** 10.3389/fimmu.2026.1835957

**Published:** 2026-06-10

**Authors:** Lin Sui, Rutie Yin

**Affiliations:** 1 Department of Obstetrics and Gynecology, West China Second Hospital of Sichuan University, Chengdu, Sichuan, China; 2 Key Laboratory of Birth Defects and Related Diseases of Women and Children, Ministry of Education, Sichuan University, Chengdu, Sichuan, China

**Keywords:** bibliometric analysis, cervical cancer, HIV-associated cervical cancer, human papillomavirus, women living with HIV (WLWH)

## Abstract

**Background:**

Research on cervical cancer among WLWH represents a critical area for advancing long-term prevention and clinical management strategies. Yet as the volume of published literature in this field continues to grow rapidly, synthesizing and appraising the available evidence in a comprehensive manner has become increasingly challenging.

**Objective:**

This study aims to map the global research landscape, identify leading contributors and collaborative networks, and delineate the knowledge base and emerging research priorities in HIV-associated cervical cancer through a bibliometric analysis of multiple databases. Particular attention is given to the molecular and immunological mechanisms underlying the synergistic oncogenic interaction between HIV and high-risk HPV, as well as current trends in translational clinical research.

**Methods:**

Publications on HIV and cervical cancer from January 1, 1990, to December 31, 2025 (n = 6,137) were retrieved and analyzed using bibliometric and visualization tools, including R (bibliometrix), Python, VOSviewer, and CiteSpace. Analyses encompassed publication trends, collaboration networks, keyword co-occurrence, and thematic evolution.

**Results:**

Research output has increased steadily, led by the United States, followed by China, South Africa, India, and the United Kingdom. The most productive journals include International Journal of Cancer and AIDS, which are also among the most frequently cited. The seminal reference is “Cancer-related Inflammation.” Emerging research frontiers center on cervical cancer screening, WLWH, human papillomavirus vaccination, and deep learning.

**Conclusions:**

Future directions will likely emphasize lifecycle vaccination strategies for WLWH, precision therapies guided by the immune microenvironment, and scalable screening approaches for low-resource settings. These findings provide evidence-based insights to inform global health policy and accelerate cervical cancer elimination efforts.

## 1 Introduction

Cervical cancer remains a major global public health challenge and is among the leading causes of cancer incidence and mortality in women worldwide (1). Despite the widespread implementation of human papillomavirus (HPV) vaccination and screening programs, the burden of cervical cancer among women living with HIV (WLWH) remains disproportionately high (2, 3). HIV-induced immune dysregulation compromises immunosurveillance, facilitating persistent infection with high-risk HPV genotypes and accelerating the progression of precancerous lesions (4). Compared with HIV-negative women, those living with HIV face a nearly six-fold increase in cervical cancer risk (5). With the global scale-up of combination antiretroviral therapy (cART), life expectancy among people living with HIV has improved substantially (6), and non-AIDS-defining cancers (NADCs) such as cervical cancer have emerged as increasingly important determinants of long-term survival and quality of life in this population. A pronounced imbalance persists, however, between the distribution of research resources and the actual burden of disease. Low- and middle-income countries — particularly those in sub-Saharan Africa — bear a dual burden of high HIV prevalence alongside inadequate screening infrastructure and limited investment in clinical research (1). Mapping the current state of research in this field is therefore essential to inform more equitable resource allocation and the development of targeted prevention and control strategies.

The pathogenesis of cervical cancer in the context of HIV involves more than immunosuppression alone. A growing body of mechanistic work points to a synergistic interplay between the two viruses that fundamentally alters the trajectory of cervical carcinogenesis (7). The HPV oncoproteins E6 and E7 disable p53 and pRb, respectively, thereby subverting cell-cycle control, apoptotic responses, and genomic stability (8). HIV amplifies this process at several levels: the Tat protein has been shown to transactivate the HPV long control region, upregulating E6 and E7 expression (9, 10), while Nef-mediated downregulation of MHC class I impairs antigen presentation and weakens CD8^+^ T-cell immune surveillance in the HPV/HIV coinfection milieu (11, 12). These intracellular events operate alongside broader immunological changes — notably, HIV-driven depletion of cervical CD4^+^ T cells and sustained mucosal inflammation — that together create a local environment permissive to HPV persistence and neoplastic progression (13, 14).

Despite the growing body of clinical and mechanistic research on HIV-associated cervical cancer systematic and quantitative evaluations of the global scientific landscape remain limited. Conventional reviews, often focused on specific clinical questions, fail to capture collaboration networks, evolving trends, and the underlying knowledge structure. Leveraging bibliometric approaches and integrating data from multiple major databases, this study provides a comprehensive and robust analysis of HIV-associated cervical cancer research from 1990 to 2025. We aim to identify key contributors, research hotspots, and emerging trends, thereby offering evidence-based insights to inform global health policy and future research priorities.

## 2 Materials and methods

### 2.1 Literature search and selection strategy

**Figure 1:**
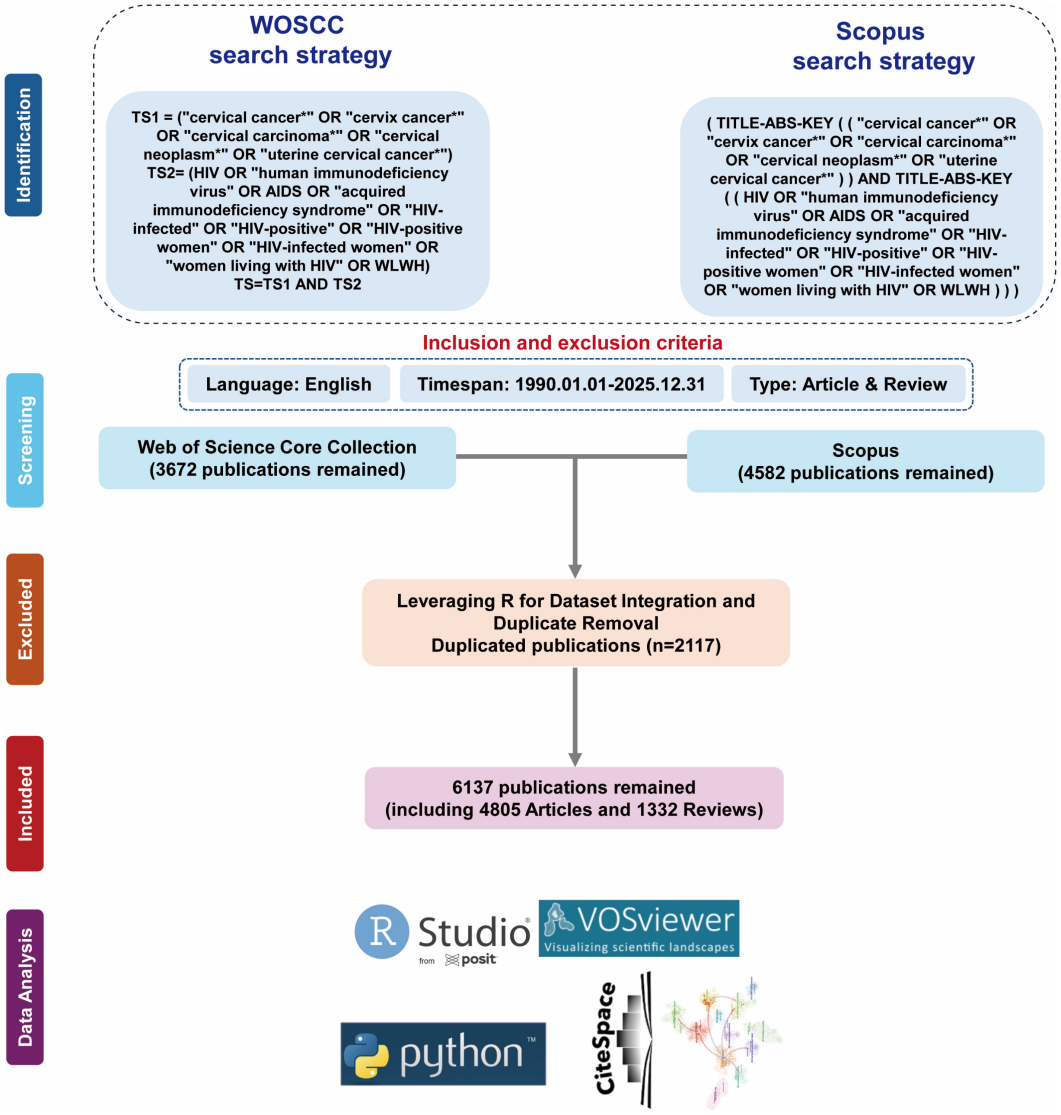
PRISMA flow diagram depicting the literature screening and inclusion process across multiple databases. This diagram illustrates the identification, screening, eligibility, and inclusion steps, ultimately resulting in the final dataset used for bibliometric analysis.

A systematic literature search was conducted on January 29, 2026, using the Web of Science Core Collection (WoSCC) and Scopus databases. The search covered publications from January 1, 1990, to December 31, 2025. Query strategies combined terms related to cervical cancer (e.g., “cervical carcinoma,” “cervix cancer”) and human immunodeficiency virus (e.g., “HIV,” “acquired immunodeficiency syndrome”). In WoSCC, searches were restricted to the Topic field (TS), encompassing titles, abstracts, and keywords, whereas in Scopus, the TITLE-ABS-KEY field was applied. Only English-language articles and reviews were included. Detailed search strategies and database characteristics are provided in **Supplementary Table 1**. Study selection followed the PRISMA framework (**Figure 1**). The initial search yielded 3,672 records from WoSCC and 4,582 from Scopus. Records were subsequently integrated and deduplicated using the bibliometrix package in R. Text normalization—including removal of non-alphabetic characters, punctuation, and extraneous whitespace—was applied to minimize formatting inconsistencies. Duplicate entries were then identified based on titles, authors, and publication years. A total of 2,117 duplicates were removed, resulting in 6,137 unique publications for downstream analysis, including 4,805 original articles and 1,332 reviews. Titles, abstracts, and keywords of all included records constituted the analytical corpus.

### 2.2 Data analysis and visualization

A multidimensional bibliometric framework was employed to quantitatively characterize the field, encompassing geographic distribution, collaboration networks, institutional contributions, journal co-occurrence, and citation structures, drawing on visualization strategies previously applied to cervical cancer–related research (15). Descriptive analyses were performed using the bibliometrix package in R (v4.4.5; v5.0), including annual publication trends, core author identification, journal distribution, collaboration patterns, and citation metrics. The package was further used to extract network matrices and construct thematic evolution maps, enabling visualization of spatiotemporal research dynamics. Network visualization and clustering were conducted using VOSviewer (v1.6.20), applying the Fruchterman–Reingold layout algorithm and association strength normalization to control for scale effects. Community structures were identified using the Walktrap algorithm. CiteSpace (v6.2.R1) was employed for keyword clustering, burst detection of keywords and references, with time slicing set to one-year intervals. Node selection parameters were set as follows: g-index (k = 25), LRF = 2.5, L/N = 10, LBY = 5, and e = 1.0. To ensure consistency and high-quality visualization, selected outputs were further processed in Python using pandas, matplotlib, seaborn, and networkx. These tools were used for standardized data formatting and graphical refinement.

### 2.3 Ethics and data availability

Ethics Statement: As this study was a bibliometric analysis of previously published literature, ethical approval was not required. Data Availability Statement: The data analyzed in this study are publicly available in the Web of Science Core Collection and Scopus databases.

## 3 Results

### 3.1 General characteristics

The three datasets, WoSCC, Scopus, and their integrated counterpart (WoSCC + Scopus), demonstrate complementary strengths in literature coverage. The merged dataset yields 6,137 publications, substantially exceeding WoSCC with 3,672 and Scopus with 4,582, highlighting the value of multi-source integration in maximizing retrieval. Original articles predominate, numbering 4,805, alongside 1,332 reviews, reflecting a field supported by both extensive empirical research and mature knowledge synthesis (**Table 1****;** **Supplementary Figure 1****).**

**Table 1:** Comparative overview of dataset characteristics across Web of Science, Scopus, and the combined dataset.

Description	WoSCC	Scopus	WoSCC+Scopus
Timespan	1990:2025	1990:2025	1990:2025
Sources (journals, books, etc)	990	1302	1692
Documents	3672	4582	6137
Annual growth rate %	11.26	10.43	10.45
Document average age	10.6	11.4	11.2
Average citations per doc	33.95	42.41	40.17
References	111156	24387	125981
Keywords plus (ID)	5646	17386	19256
Author’s keywords (DE)	5700	5858	8329
Authors	19298	20550	30679
Authors of single-authored docs	140	296	364
Single-authored docs	160	401	461
Co-authors per doc	7.14	6.96	6.81
International co-authorships %	39	36.1	33.96
Article	3055	3483	4805
Review	617	1099	1332

All datasets span 1990–2025, with stable annual growth rates of 10.4%–11.3%. WOSCC shows a marginally higher rate (11.26%), suggesting more rapid recent expansion. The mean publication age (10.6–11.4 years) indicates that current research primarily builds on evidence from the past decade, consistent with a moderately evolving knowledge base. As illustrated in **Supplementary Figure 1**, the merged dataset maintains the highest cumulative output across the entire period.

In terms of impact, Scopus-indexed publications achieve the highest average citations per article (42.41), exceeding WoSCC (39.95) and the merged dataset (40.17), likely reflecting broader journal inclusion (**Table 1****;** **Supplementary Figure 1**). However, the merged dataset leads in total references (125,981), author keywords (8,329), and collaboration scale (30,679 authors), indicating stronger knowledge integration and collaborative potential.

Collaboration analysis shows a mean of 6.81–7.14 authors per paper, with international co-authorship rates ranging from 33.96% to 39%, highest in WoSCC. Single-authored studies remain rare (7.5% in the merged dataset), underscoring the dominance of collaborative research in this field.

### 3.2 Publication trends and geographic distribution

**Figure 2:**
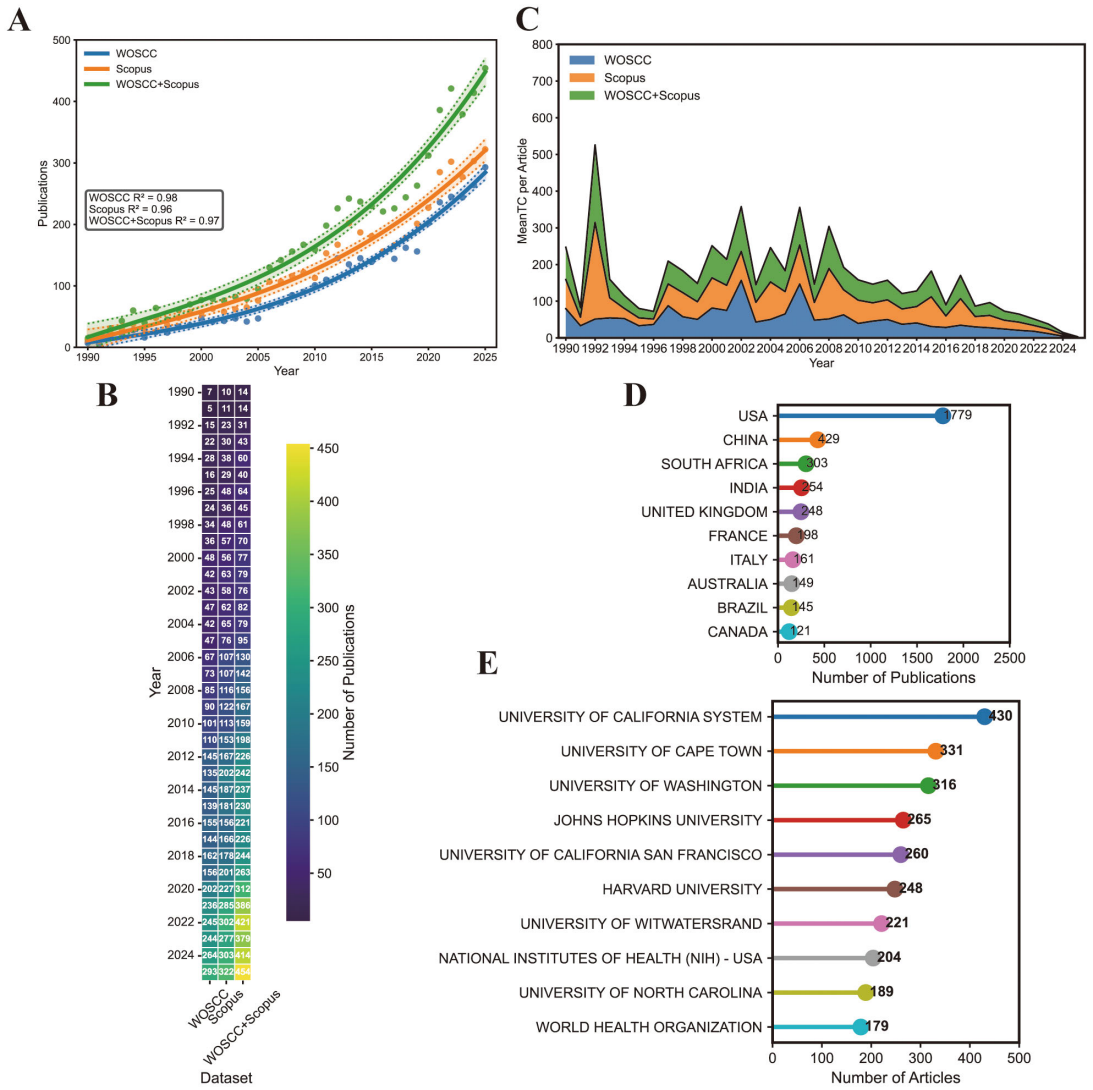
Publication trends and geographic distribution. **(A)** Annual publication trends with fitted curves across Web of Science Core Collection, Scopus, and the combined dataset. **(B)** Quantitative comparison of publication output across the datasets. **(C)** Annual citation counts for publications in each dataset. **(D)** Lollipop chart showing the top ten countries by publication volume in the combined dataset. **(E)** Lollipop chart illustrating the top ten institutions by publication volume in the combined dataset.

From 1990 to 2025, research on cervical cancer–HIV comorbidity demonstrated a clear exponential growth pattern. High goodness-of-fit values (R^2=^ 0.98 for WoSCC, 0.96 for Scopus, and 0.97 for WoSCC + Scopus) confirm the robustness of this trend. The merged dataset not only yielded the highest publication volume but also exhibited a marked post-2010 acceleration, reflecting intensifying scholarly attention to this interdisciplinary field. Although Scopus slightly outperformed WoSCC in absolute output, their growth trajectories have become increasingly aligned over time (**Figures 2A, B**).

Citation patterns varied substantially across years. Earlier publications achieved notably higher per-article impact; for example, those from 1992 reached an average of 211.32 citations, whereas more recent studies remain in the early phase of citation accumulation. In selected years such as 2002, 2004, 2008, 2015, and 2017, Scopus-indexed articles demonstrated higher average citation rates than those in WoSCC, likely reflecting broader source coverage (**Figure 2C****;** **Supplementary Table 2**).

Geographically, a “dominant leader with multiple strong contributors” pattern is evident. The United States leads by a wide margin with 1,779 publications, accounting for 29%, followed by China, South Africa, and India. Several countries exhibit high levels of international collaboration, with MCP rates consistently approaching or exceeding 45%. Notable examples include South Africa, France, the United Kingdom, and Canada. South Africa stands out as both a high-output and highly collaborative country, highlighting the influence of disease burden on research activity and the importance of global partnerships (**Figure 2D****;** **Table 2**).

**Table 2:** Top ten countries by publication output and their collaboration rates.

Country	Articles	Articles %	SCP	MCP	MCP %
USA	1779	29	1175	604	34
CHINA	429	7	356	73	17
SOUTH AFRICA	303	4.9	162	141	46.5
INDIA	254	4.1	198	56	22
UNITED KINGDOM	248	4	129	119	48
FRANCE	198	3.2	98	100	50.5
ITALY	161	2.6	127	34	21.1
AUSTRALIA	149	2.4	89	60	40.3
BRAZIL	145	2.4	116	29	20
CANADA	121	2	65	56	46.3

SCP (Single-Country Publications) refers to articles in which all authors are affiliated with institutions in the same country; MCP (Multiple-Country Publications) refers to articles involving authors from two or more countries. MCP% indicates the proportion of internationally collaborative publications among a country’s total output, reflecting the degree of cross-border research engagement.

At the institutional level, the University of California system ranks first, followed by the University of Cape Town and the University of Washington. Half of the top ten institutions are based in the United States, alongside key contributors such as Johns Hopkins University and Harvard University. Other prominent institutions include the University of the Witwatersrand, the National Institutes of Health, the University of North Carolina, and the World Health Organization. Overall, research capacity is concentrated in countries with either strong public health infrastructures or high epidemiological burden, forming a globally interconnected landscape led by the United States and reinforced by active international collaboration (**Figure 2E**).

### 3.3 Author, institutional, and journal productivity

**Figure 3:**
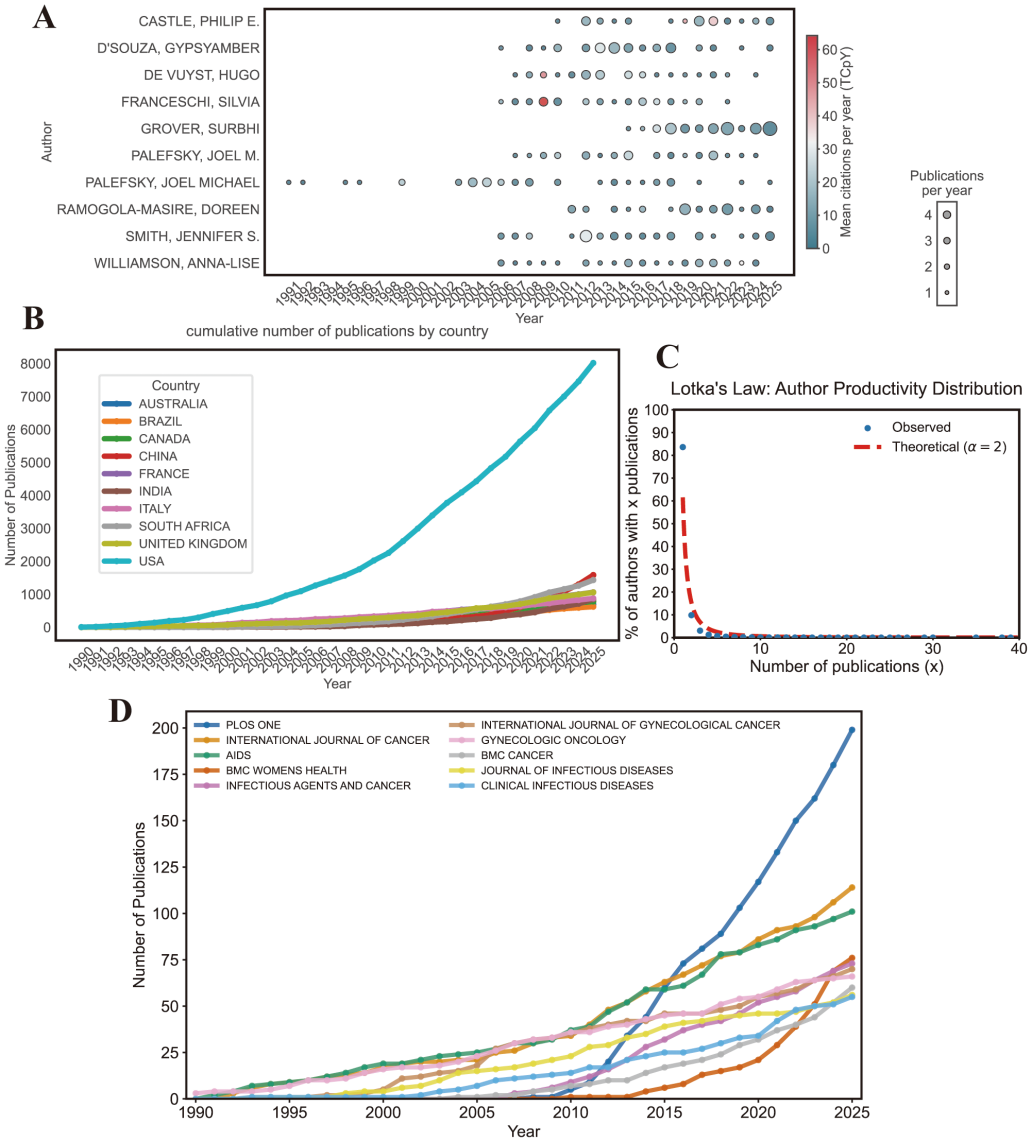
Author, institution, and journal productivity analysis. **(A)** Yearly publication output of the top ten authors. **(B)** Yearly publication output of the top ten countries. **(C)** Author productivity distribution according to Lotka’s Law. **(D)** Yearly publication output of the top ten journals.

Among all authors, Franceschi Silvia ranks foremost with 30 publications and 2,882 total citations. She also holds the highest H-index (16) and M-index (1.143), reflecting sustained and influential scholarly contributions. Palefsky Joel Michael has the greatest publication output (n = 38), though his earlier entry into the field (since 1991) results in a comparatively lower M-index (0.639). Nevertheless, his G-index of 38 underscores a substantial number of highly cited works. D’Souza Gypsyamber follows closely with 42 publications, indicative of notable research activity (**Figure 3A****;** **Table 3**).

**Table 3:** Top ten authors ranked by academic productivity metrics.

Author	h_index	g_index	m_index	TC	NP	PY_start
FRANCESCHI SILVIA	24	30	1.143	2882	30	2006
PALEFSKY JOEL MICHAEL	23	38	0.639	2795	38	1991
SAHASRABUDDHE VIKRANT V.	20	25	0.952	1419	25	2006
SMITH JENNIFER S.	20	38	0.952	1493	39	2006
WILLIAMSON ANNA-LISE	19	30	0.905	1079	30	2006
D’SOUZA GYPSYAMBER	18	38	0.857	1451	42	2006
DE VUYST HUGO	18	29	0.9	2359	29	2007
DENNY LYNETTE	18	26	0.857	1401	26	2006
CASTLE PHILIP E.	16	29	0.941	1238	29	2010
CLIFFORD GARY M.	16	20	0.762	2774	20	2006

h_index, number of papers each cited at least *h* times; g_index, a citation metric that gives more weight to highly cited papers; m_index, h_index divided by academic career length in years; TC, total citations; NP, number of publications; PY_start, year of first publication in the dataset.

Lotka’s law analysis further demonstrates that author productivity conforms to an inverse-square distribution, whereby the vast majority of researchers contribute only a single publication, while a small core group accounts for a disproportionate share of output. This pattern suggests a highly dispersed authorship structure, with a relatively limited and not yet fully consolidated core author network (**Figure 3C**). At the national level, the United States consistently leads in publication volume with a substantial margin, while China has exhibited rapid growth in recent years, firmly establishing itself in second place. High-burden countries such as South Africa and India also show sustained increases in output, reflecting a globally heightened research focus on this public health challenge (**Figure 3B**).

Journal distribution analysis identifies International Journal of Cancer as the leading publication venue, with 114 articles, 10,180 total citations, and an H-index of 47; its M-index (1.343) further indicates enduring academic influence. AIDS and PLOS ONE rank second and third, respectively. Notably, despite its relatively recent inception (2008), PLOS ONE exhibits the highest M-index (2.0) alongside a substantial publication volume (n = 199), underscoring its growing prominence as a dissemination platform. Established infectious disease journals, including Journal of Infectious Diseases and Clinical Infectious Diseases, also feature prominently, highlighting the disciplinary foundation of this research domain (**Table 4**).

**Table 4:** Top ten journals ranked by academic productivity metrics.

Source	h_index	g_index	m_index	TC	NP	PY_start
INTERNATIONAL JOURNAL OF CANCER	47	100	1.343	10180	114	1992
AIDS	42	82	1.167	6911	101	1991
PLOS ONE	38	60	2	4947	199	2008
JOURNAL OF INFECTIOUS DISEASES	31	56	0.912	3779	56	1993
CLINICAL INFECTIOUS DISEASES	30	55	0.882	4104	55	1993
GYNECOLOGIC ONCOLOGY	28	51	0.757	2762	66	1990
INFECTIOUS AGENTS AND CANCER	25	39	1.25	1777	73	2007
VACCINE	23	42	0.719	4535	42	1995
BMC PUBLIC HEALTH	22	35	1.158	1296	43	2008
SEXUALLY TRANSMITTED DISEASES	22	42	0.611	1792	47	1991

h_index, g_index, m_index, TC, NP, and PY_start are defined as in **Table 3**, here applied at the journal level rather than the author level.

Collectively, the top ten journals demonstrate a steady increase in annual publication output over time, with a marked acceleration after 2010, indicative of the expanding academic engagement and the increasing importance of dedicated platforms for this interdisciplinary field (**Figure 3D**).

### 3.4 Keyword analysis

**Figure 4:**
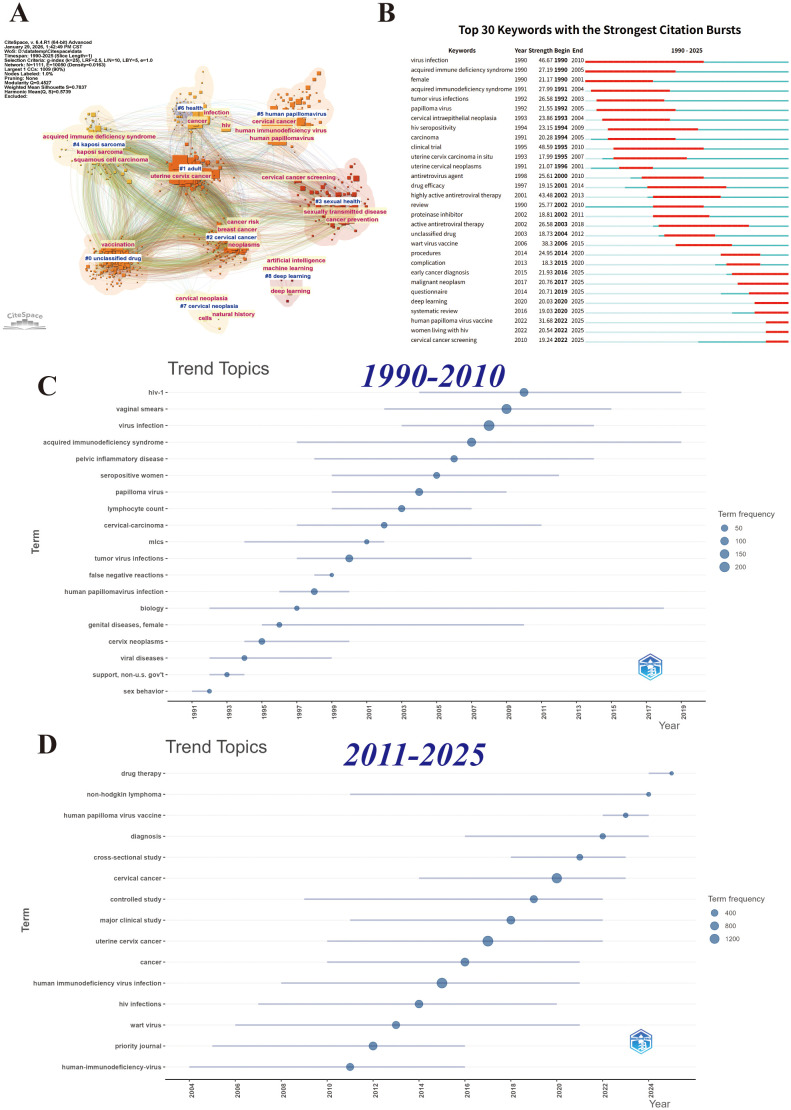
Keyword analysis. **(A)** Network clustering of high-frequency keywords. **(B)** Top 30 keywords with the strongest citation bursts. **(C)** Keyword trend analysis for years 1990–2010. **(D)** Keyword trend analysis for years 2011–2025.

CiteSpace-based keyword co-occurrence and clustering identified nine major clusters (Modularity Q = 0.4527; Silhouette S = 0.7837), delineating the knowledge structure of cervical cancer–HIV comorbidity research. Core clusters such as “adult,” “cervical cancer,” and “sexual health” highlight a primary focus on adult women, the disease itself, and its connection to sexual health. Clusters including “human papillomavirus” and “infection” underscore the central etiological role of HPV, while “cervical neoplasia,” “deep learning,” and “unclassified drug” reflect a shift from foundational pathology toward emerging domains, including AI-assisted diagnostics and novel therapeutics (**Figure 4A**).

High-frequency keywords are both concentrated and thematically coherent. “Human immunodeficiency virus” (n = 1,620), “cervical cancer” (n = 1,468), and “human papillomavirus” (n = 1,126) form the conceptual core of the field. The prominence of “women,” “infection,” and “neoplasms” further emphasizes a focus on female populations and virus-driven oncogenesis. Meanwhile, terms such as “prevalence,” “risk,” and “screening” highlight the central role of epidemiological assessment and early detection (**Supplementary Table 3**).

Burst detection captures the evolution of research priorities. Early studies (1990s–2000s) centered on virology and epidemiology (e.g., “virus infection,” “acquired immunodeficiency syndrome”). In the mid-phase (2000s–2010s), attention shifted toward antiretroviral therapy and precancerous lesions (e.g., “highly active antiretroviral therapy,” “cervical intraepithelial neoplasia”). Since 2015, emerging terms such as “deep learning,” “systematic review,” and “human papillomavirus vaccine” indicate a transition toward precision medicine, methodological consolidation, and technological integration (**Figure 4B**).

Trend analysis further confirms this shift. From 1990 to 2010, research emphasized descriptive and mechanistic themes (e.g., “HIV-1,” “vaginal smears”). In contrast, the 2011–2025 period is characterized by a focus on intervention and population-level management, including “drug therapy,” vaccination, and large-scale epidemiological studies, marking a transition toward comprehensive prevention and control strategies (**Figures 4C, D**).

### 3.5 Comparative validation of collaboration networks

To characterize global collaboration patterns, co-authorship networks at the country, author, and institutional levels were constructed using both WoSCC and Scopus, enabling cross-database validation of structural consistency.

**Figure 5:**
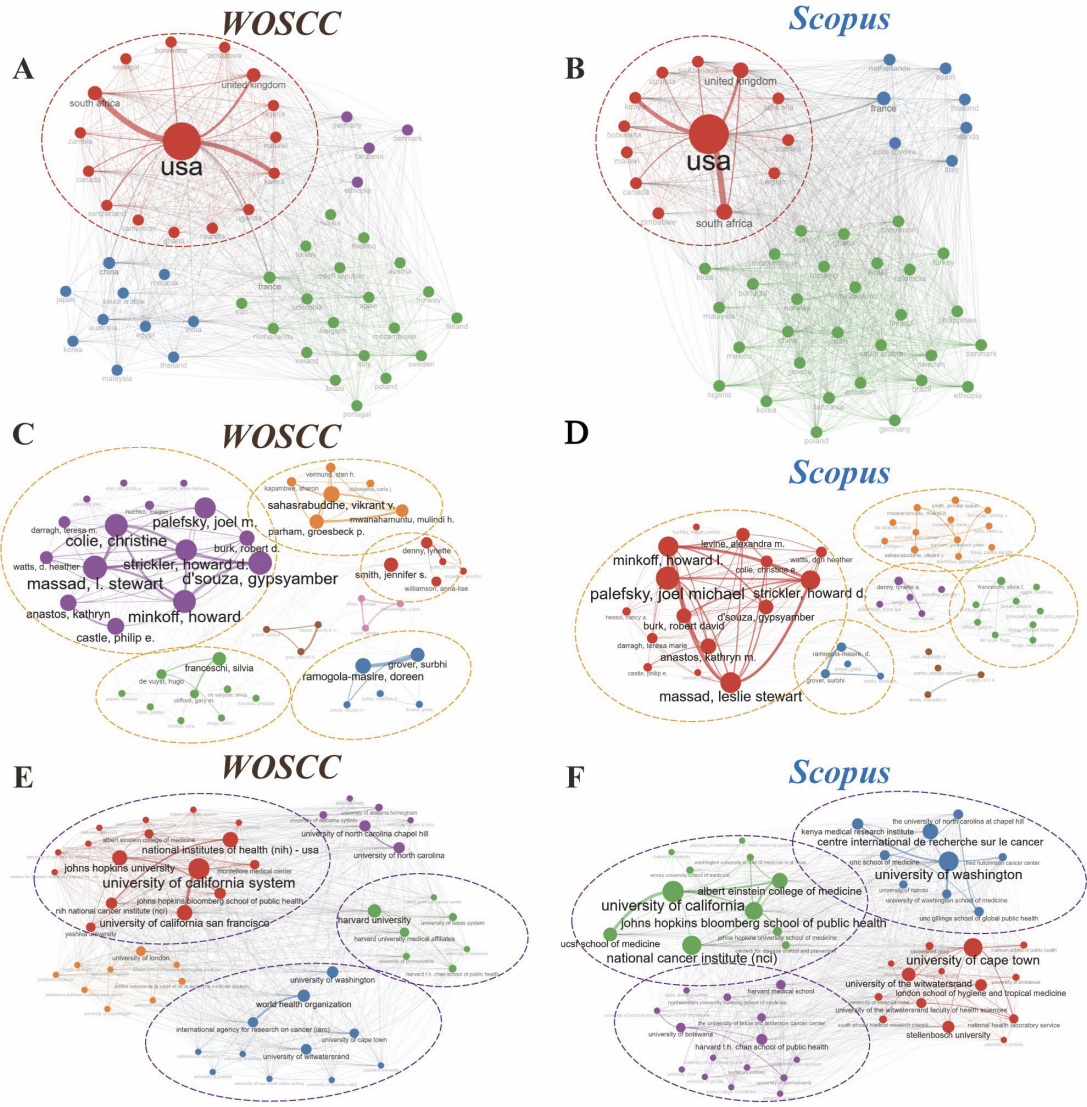
Comparative analysis of collaboration networks among authors, countries, and institutions. **(A, B)** Author collaboration networks from Web of Science and Scopus. **(C, D)** Country-level collaboration networks from Web of Science and Scopus. **(E, F)** Institutional collaboration networks from Web of Science and Scopus.

At the country level (**Figures 5A, B**), the United States consistently occupies a central and dominant position, with the largest node size and strongest connectivity. It forms a core cluster and maintains close ties with countries such as South Africa, the United Kingdom, and Canada. Additional regional subnetworks, such as those in Europe and Asia, are more prominent in Scopus, but their linkage to the United States remains consistent across both databases, supporting a globally coordinated, U.S.-led structure. At the author level (**Figures 5C, D**), both datasets identify overlapping core research groups. Key authors, including Palefsky Joel M., D’Souza Gypsyamber, and Strickler Howard D., consistently occupy central positions, indicating stable collaborative communities.

At the institutional level (**Figures 5E, F**), major hubs such as the National Institutes of Health, the University of California system, Johns Hopkins University, and Harvard University anchor extensive cross-institutional networks in both databases.

Overall, the collaboration structures derived from WoSCC and Scopus are highly concordant, with consistent core nodes, clusters, and linkages, supporting the robustness and reliability of the observed collaboration patterns.

### 3.6 Citation analysis

**Figure 6:**
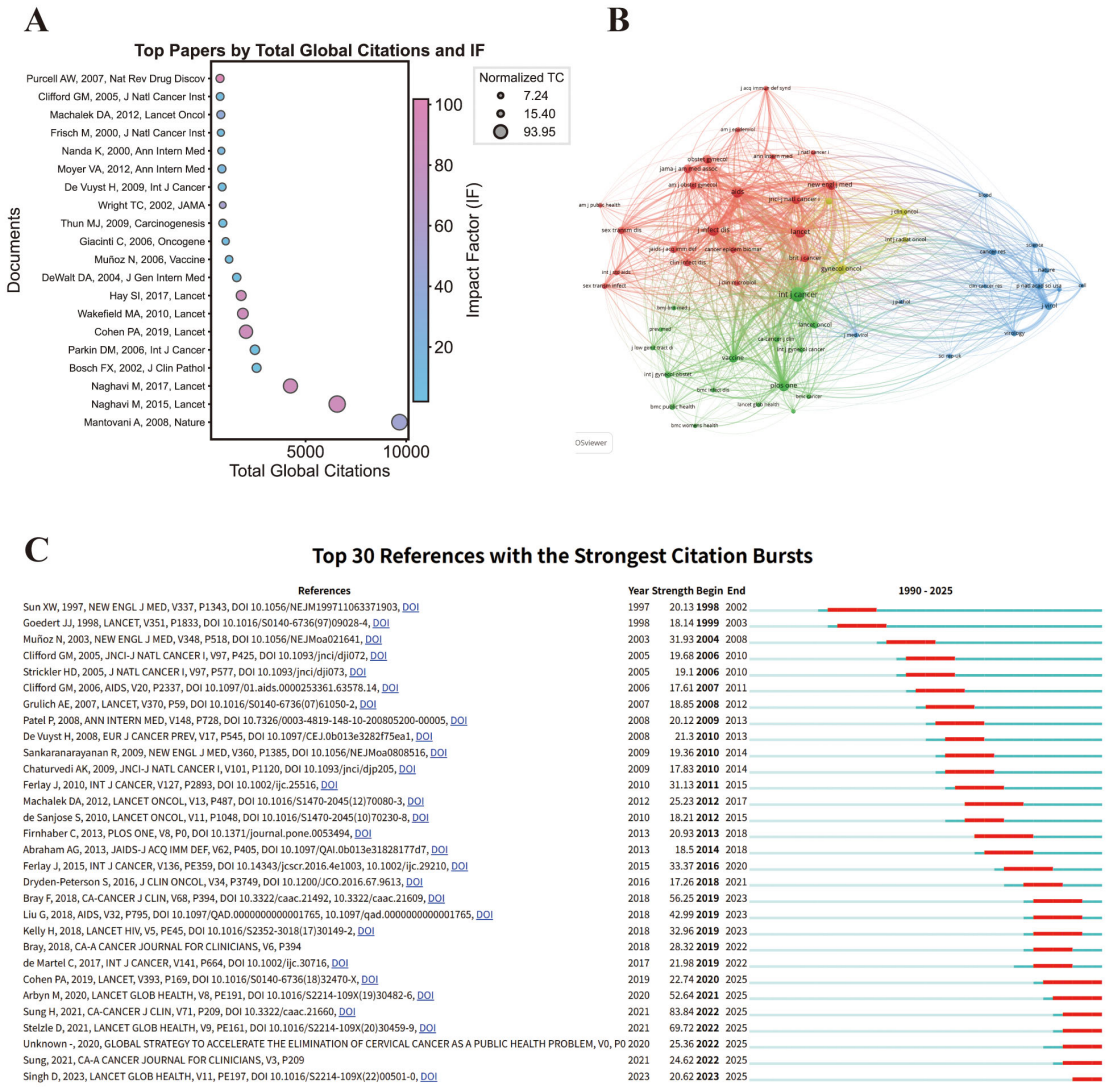
Citation analysis. **(A)** Top 20 globally cited publications visualized as a bubble chart with citation counts and journal impact factors. **(B)** Co-cited journals network visualized using VOSviewer. **(C)** Top 30 publications with the strongest citation bursts.

The most highly cited publication in the field is Mantovani A (2008, *Nature*), with 9,669 total citations, an annual citation rate of 508.89, and a normalized citation score of 84.46. Close followers are two landmark studies on global disease burden by Naghavi M (2015, 2017, *The Lancet*), with 6,570 and 4,248 total citations, respectively. Among the top twenty cited articles, five were published in *The Lancet*, three in *International Journal of Cancer*, two in *Annals of Internal Medicine*, and the remainder in high-impact journals including *JAMA*, *Nature Reviews Drug Discovery*, and *Oncogene*. Citation counts range from 749 to 9,669, while journal impact factors vary widely (2.0–101.8), with Purcell AW (2007) appearing in the highest-impact journal (**Figure 6A****;** **Table 5**).

**Table 5:** Top 20 globally cited publications with detailed information.

Paper	DOI	Total citations	TC per year	Normalized TC	IF
MANTOVANI A, 2008, NATURE	10.1038/nature07205	9669	508.89	84.46	48.5
NAGHAVI M, 2015, LANCET	10.1016/S0140-6736(14)61682-2	6570	547.50	93.95	88.5
NAGHAVI M, 2017, LANCET	10.1016/S0140-6736(17)32152-9	4248	424.80	67.45	88.5
BOSCH FX, 2002, J CLIN PATHOL	10.1136/jcp.55.4.244	2565	102.60	21.04	2
PARKIN DM, 2006, INT J CANCER	10.1002/ijc.21731	2483	118.24	24.23	4.7
COHEN PA, 2019, LANCET	10.1016/S0140-6736(18)32470-X	2040	255.00	58.27	88.5
WAKEFIELD MA, 2010, LANCET	10.1016/S0140-6736(10)60809-4	1884	110.82	34.09	88.5
HAY SI, 2017, LANCET	10.1016/S0140-6736(17)32130-X	1800	180.00	28.58	88.5
DEWALT DA, 2004, J. GEN. INTERN. MED.	10.1111/j.1525-1497.2004.40153.x	1579	68.65	16.93	4.2
MUÑOZ N, 2006, VACCINE	10.1016/j.vaccine.2006.05.115	1200	57.14	11.71	3.5
GIACINTI C, 2006, ONCOGENE	10.1038/sj.onc.1209615	1031	49.10	10.06	7.3
THUN MJ, 2009, CARCINOGENESIS	10.1093/carcin/bgp263	888	49.33	14.54	2.9
WRIGHT TC, 2002, JAMA-J AM MED ASSOC	10.1001/jama.287.16.2120	883	35.32	7.24	55
DE VUYST H, 2009, INT J CANCER	10.1002/ijc.24116	854	47.44	13.98	4.7
MOYER VA, 2012, ANN INTERN MED	10.7326/0003-4819-156-12-201206190-00424	840	56.00	15.77	15.2
NANDA K, 2000, ANN INTERN MED	10.7326/0003-4819-132-10-200005160-00009	813	30.11	9.30	15.2
FRISCH M, 2000, J. NATL. CANCER INST.	10.1093/jnci/92.18.1500	791	29.30	9.05	7.2
MACHALEK DA, 2012, LANCET ONCOL	10.1016/S1470-2045(12)70080-3	789	52.60	14.82	35.9
CLIFFORD GM, 2005, JNCI-J NATL CANCER I	10.1093/jnci/dji072	755	34.32	13.28	7.2
PURCELL AW, 2007, NAT. REV. DRUG DISCOV.	10.1038/nrd2224	749	37.45	15.03	101.8

TC per Year, total citations divided by the number of years since publication; Normalized TC, citation count adjusted relative to the average citation rate of all papers published in the same year, allowing cross-year comparison; IF, journal impact factor.

At the journal level, *International Journal of Cancer* leads with 5,480 co-citations and a total link strength of 169,101, highlighting its central role in knowledge dissemination. It is followed by *AIDS* (3,529), *The Lancet* (3,380), *Journal of Infectious Diseases* (3,351), and *PLOS ONE* (3,231). Top-cited journals collectively span oncology (e.g., *Gynecologic Oncology*, *British Journal of Cancer*, *Lancet Oncology*), infectious disease (e.g., *Clinical Infectious Diseases*, *JAIDS*), and general medicine (e.g., *New England Journal of Medicine*, *JAMA*). High total link strength values (generally >50,000) indicate dense co-citation networks, forming a tightly interconnected scholarly community (**Figure 6B****;** **Table 6**).

**Table 6:** Top 20 co-cited journals visualized using VOSviewer.

Source	Citations	Total link strength
Int J Cancer	5480	169101
AIDS	3529	118643
Lancet	3380	120280
J Infect Dis	3351	125093
PLoS One	3231	88568
Gynecol Oncol	2664	76870
N Engl J Med	2614	99809
J Natl Cancer Inst	2227	83027
Vaccine	2215	69971
JAMA	1954	65010
Obstet Gynecol	1932	60941
J Virol	1910	77288
Br J Cancer	1804	68888
Sex Transm Dis	1451	55062
Lancet Oncol	1442	52487
Clin Infect Dis	1398	50139
J Acquir Immune Defic Syndr	1385	46122
Am J Obstet Gynecol	1380	47992
Cancer Epidemiol Biomarkers Prev	1355	55760
J Clin Oncol	1253	52392

Dual-map overlay analysis using CiteSpace reveals clear patterns of knowledge flow. Citing journals cluster in applied disciplines such as medicine, clinical practice, and molecular biology/immunology, while cited sources extend from foundational biomedical research to broader domains including psychology and economics. Key trajectories indicate that foundational work in molecular biology and genetics (Z-score = 7.33) and in health, nursing, and medicine (Z-score = 6.28) has been a major driver of clinical research, as reflected in the main green pathway. A notable intra-foundational path (yellow stream, Z-score = 4.37) is also evident. Additional flows toward “psychology, education, and health” highlight the growing relevance of socio-behavioral interventions. Overall, the field has developed a biomedically anchored, interdisciplinary ecosystem integrating public health and social sciences (**Supplementary Figure 2****).**

### 3.7 Thematic evolution analysis

**Figure 7:**
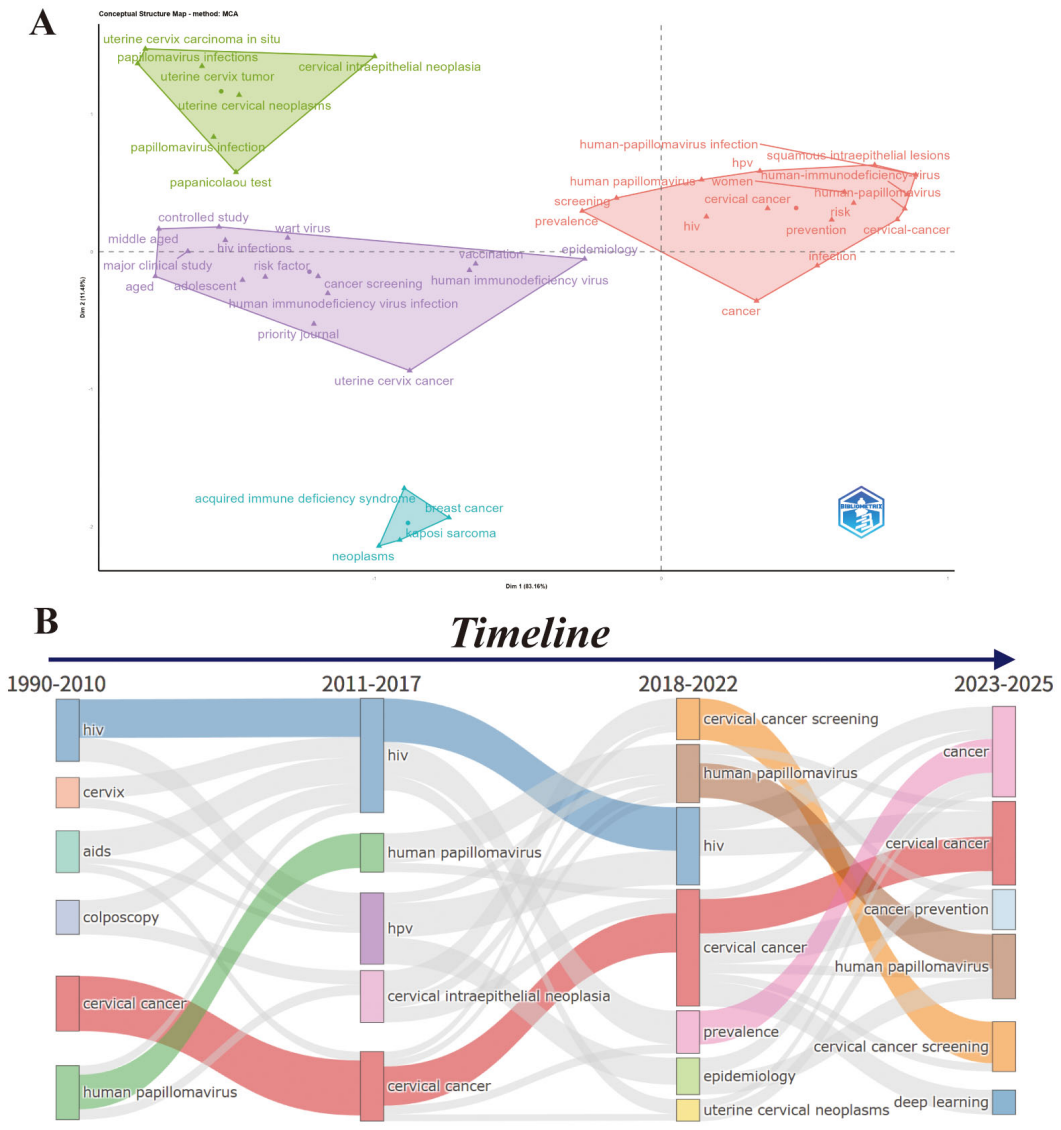
Thematic evolution analysis. **(A)** Multiple correspondence analysis illustrating relationships among research themes. **(B)** Thematic evolution map showing the development and transition of key topics over time.

Multiple correspondence analysis (MCA) and thematic stream mapping of WoSCC and Scopus datasets reveal the structural and temporal evolution of cervical cancer–HIV research (**Figure 7**). MCA identified four clusters. The upper-left cluster (green), which includes uterine cervix carcinoma *in situ*, cervical intraepithelial neoplasia, papillomavirus infections, and the Papanicolaou test, corresponds to foundational work in cytological screening and pathology. The upper-right cluster (red), encompassing human papillomavirus infection, HIV, prevalence, risk, and squamous intraepithelial lesions, reflects research on viral co-infection and risk assessment. The lower-left (purple) cluster, with “controlled study,” “major clinical study,” “human immunodeficiency virus infection,” “aged,” and “adolescent,” reflects clinical research in age-specific and immunocompromised populations. The lower (cyan) cluster, including “acquired immune deficiency syndrome,” “neoplasms,” “Kaposi sarcoma,” and “breast cancer,” signals attention to comorbid malignancies among HIV-positive individuals (**Figure 7A**).

Thematic stream mapping shows a three-phase evolution from 1990 to 2025. From 1990 to 2010, research centered on establishing foundational knowledge, with terms such as “HIV,” “AIDS,” “cervix,” and “colposcopy” emphasizing prevalence and descriptive studies. Between 2011 and 2017, mechanistic exploration intensified, as “human papillomavirus” and “HPV” became central, coupled with “cervical intraepithelial neoplasia,” marking progress toward elucidating the HIV–HPV–cancer pathogenic axis. From 2018 onward, focus shifted to prevention and technological integration: “cervical cancer screening” and “cancer prevention” dominate, while emerging terms like “deep learning” indicate AI applications in imaging and risk prediction. Throughout, “cervical cancer” and “human papillomavirus” remain foundational, underpinning comprehensive prevention and control strategies. This progression reflects a clear shift from descriptive and mechanistic studies toward intervention-driven, technology-enabled approaches (**Figure 7B**).

## 4 Discussion

This study systematically analyzed 6,137 publications on HIV-related cervical cancer from 1990 to 2025. Analysis of trends across two authoritative databases showed a marked exponential increase in scientific output over the past three decades (R² = 0.97), in line with growing public health interest in cervical cancer as a non-AIDS-defining cancer (NADC) alongside the global scale-up of combination antiretroviral therapy (cART) and the extended life expectancy of people living with HIV. Over time, research focus has progressively shifted from elucidating pathological mechanisms to advancing global elimination strategies.

In terms of national contributions, the United States has established its global leadership in this field with 1,779 publications, supported by its strong foundation in basic medical research and sustained funding through programs such as PEPFAR (President’s Emergency Plan for AIDS Relief). Meanwhile, South Africa and China, representing developing countries, have also seen significant upward trajectories in their research output. The shifting geopolitical landscape reflects several underlying factors. South Africa’s rise is closely linked to its dual burden of HIV and cervical cancer, where clinical need has directly shaped research priorities. China’s rapid growth in publications owes largely to broader gains in national research capacity, sustained public health funding, and a strategic policy focus on women’s reproductive health screening.

The main contributors among the journals publishing these academic results include *PLOS ONE*, *AIDS*, *International Journal of Cancer*, and *Journal of Infectious Diseases*. Notably, *PLOS ONE*, as a representative interdisciplinary journal, has not only contributed the largest volume of publications but also facilitated the cross-disciplinary dissemination of research findings. Meanwhile, *AIDS*, as a traditional authoritative specialty journal, continues to maintain a high citation impact, solidifying its core position in defining clinical standards and guiding academic trends.

The following discussion of topic hotspots is based on bibliometric findings, particularly the keyword co-occurrence clustering in **Figure 4**, the citation burst analysis in **Figure 6**, and the topic evolution flow in **Figure 7**. These structures clearly reveal how the research paradigm in this field has evolved from the early focus on the “biological mechanisms of HIV-HPV co-infection” to “precision public health interventions under global elimination strategies”.

### 4.1 HIV-HPV synergistic pathogenesis and immune microenvironment remodeling

This hotspot corresponds primarily to the early high-frequency terms such as “viral infection” and “immune deficiency” in **Figure 4**. While foundational mechanistic research laid the groundwork between 1990 and 2010, recent citation bursts indicate that the exploration of synergistic pathogenic mechanisms is entering a more complex and deepening phase.

**Figure 8:**
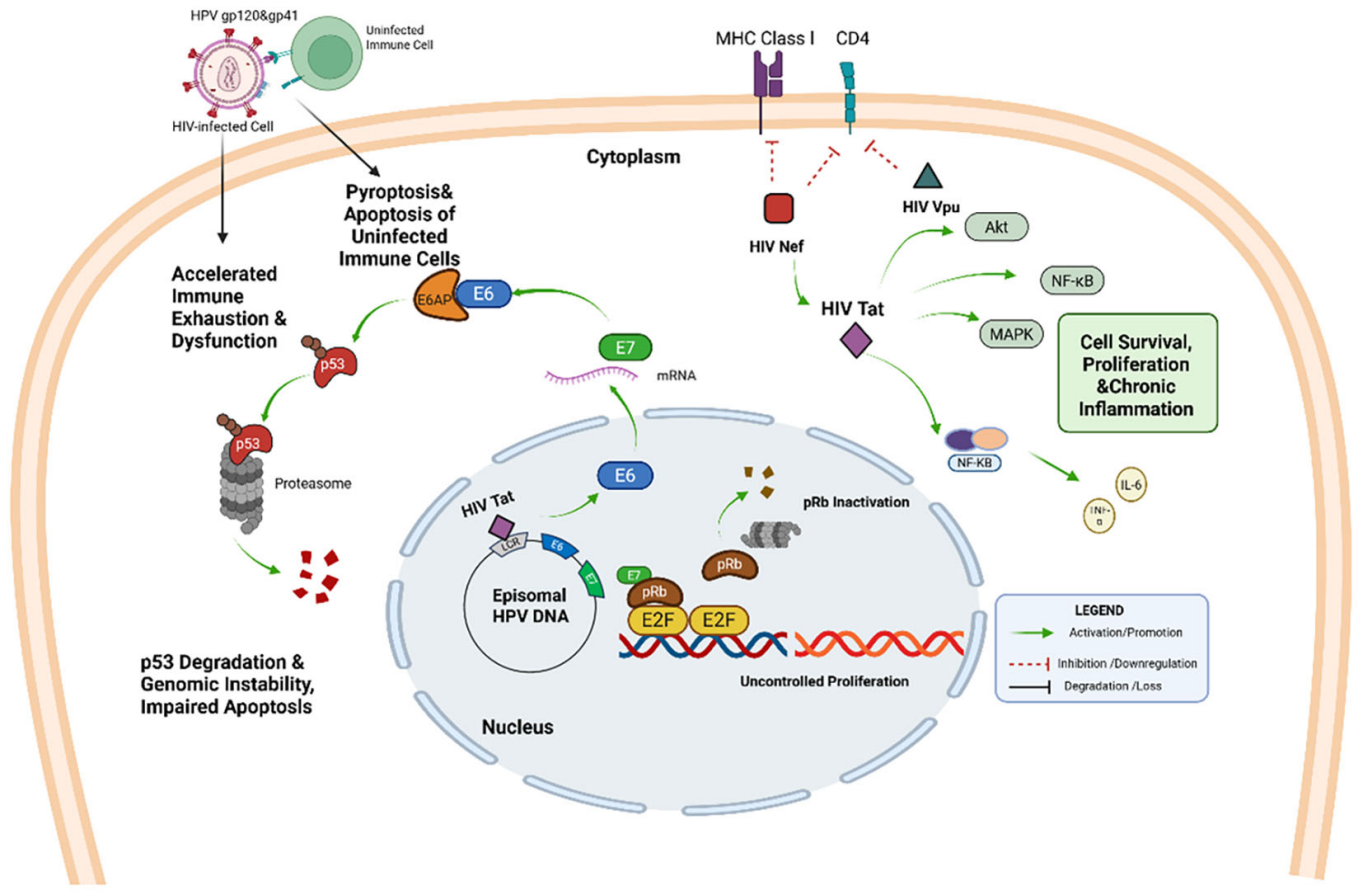
Interactions between HIV and HPV impacting cervical cancer progression: HIV Tat enhances transcription of HPV DNA, upregulating the oncogenes E6 and E7. E6, in complex with E6AP, mediates proteasomal degradation of p53, while E7 inactivates pRb and releases E2F transcription factors, collectively driving uncontrolled cellular proliferation. Concurrently, HIV Nef and Vpu downregulate MHC class I and CD4 molecules to evade immune surveillance, and Tat activates the MAPK, Akt, and NF-κB pathways to sustain chronic inflammation, thereby accelerating progression toward invasive cervical carcinoma.

Current evidence indicates that co-infection with HIV and HPV does not merely exert additive effects, but rather engages in a complex, bidirectional interplay that collectively reshapes the cervical carcinogenesis process (7, 17). HIV infection drives progressive depletion and functional exhaustion of CD4^+^ T cells, impairing antigen presentation and weakening HPV-specific CD8^+^ cytotoxic responses, thereby diminishing the clearance of infected cells. Concurrently, HIV perturbs mucosal defenses, reshapes the vaginal microbiome, and reduces lactobacilli dominance, leading to increased mucosal permeability and heightened local inflammation (3). The HPV oncoproteins E6 and E7 drive genomic instability and promote cellular proliferation by disrupting the tumor suppressor pathways governed by p53 and pRb (18). HIV co-infection amplifies this process (**Figure 8**): HIV Tat protein directly enhances HPV transcriptional activity (19), promoting the expression of E6 and E7 (7, 20), which further leads to p53 degradation and pRb inactivation, driving cell cycle dysregulation and genomic instability, thus accelerating carcinogenesis (21, 22). HIV also downregulates CD4 and MHC class I molecules through its accessory proteins Nef, Vpu, and Vif, suppressing the function of CD8^+^ T cells and impairing immune surveillance, allowing HPV-infected cells to evade immune system clearance and further promoting the persistence and spread of HPV infection (18). Furthermore, HIV activates multiple host cell signaling pathways, such as MAPK, PI3K/Akt, and NF-κB, promoting cell survival, proliferation, and chronic inflammation. Chronic inflammation, through cytokines such as IL-6 and TNF-α, further accelerates the progression of cervical cancer. Recent evidence on the inflammation-to-tumor transition mechanism further supports this pathogenic axis (23). These interactions collectively drive cervical cancer development, enhance HPV’s carcinogenic potential, and exacerbate cell proliferation and genomic instability (24). Understanding the complex mechanisms of HIV-HPV synergy is crucial for cervical cancer screening and treatment, and provides new directions for developing comprehensive treatment strategies targeting HIV/HPV co-infection in the future.

Beyond direct intracellular interactions—such as HIV Tat-mediated enhancement of HPV transcription and Nef/Vpu-induced MHC-I downregulation—HIV profoundly remodels the cervical tissue immune microenvironment (TIME), serving as a critical driver of persistent HPV infection. Chronic HIV infection shifts the local cervical microenvironment from an immune-activated state toward an immunosuppressive, tolerogenic phenotype, driven in large part by aberrant infiltration and expansion of regulatory T cells (Tregs) and myeloid-derived suppressor cells (MDSCs). HIV-associated systemic inflammation and local chemokine secretion (e.g., CCL22, CCL20) further facilitate the recruitment of CD4^+^CD25^+^FoxP3^+^ Tregs (16). These cells secrete high levels of inhibitory cytokines, including TGF-β and IL-10, which directly suppress HPV-specific CD8^+^ cytotoxic T lymphocyte (CTL) responses (25).

Concurrently, MDSC expansion in the cervical mucosa depletes key amino acids through arginase-1 (Arg-1) and inducible nitric oxide synthase (iNOS), further impairing T cell proliferation and function (26). Together, this synergistic immune subversion — compounded by upregulation of immune checkpoints such as PD-1/PD-L1 and subsequent T cell exhaustion — creates a highly permissive tolerogenic niche. This compromised immune barrier facilitates HPV immune evasion, accelerates viral persistence, and significantly expedites the progression of cervical carcinogenesis in co-infected individuals.

### 4.2 Prevention and screening strategies

With the widespread adoption of cART, the life expectancy of individuals living with HIV has significantly increased, and cervical cancer has become a major complication affecting their long-term survival. The clinical management theme closely aligns with the “Integrated Medicine/Infectious Diseases” cluster (red cluster) in **Figure 7**, as well as the most prominent keywords in **Figure 4B**, such as “Women Living with HIV” (strength 20.54) and “HPV vaccine” (strength 31.68). This shift marks a transition in research focus from mere “AIDS survival” to managing the quality of life during the “chronic disease survival phase”.

HPV vaccination represents a primary preventive strategy against cervical cancer. However, WLWH face distinct virological challenges. Notably, the distribution of HPV types in WLWH may differ from that in the general population, with a relatively lower prevalence of HPV16 in precancerous lesions. This difference could potentially diminish the overall protective efficacy of current vaccines (27, 28). Second, low CD4^+^ T cell counts may limit the vaccine’s effectiveness. Nevertheless, multiple clinical studies have confirmed that the HPV vaccine is safe and immunogenic in HIV-infected populations (29). Studies have shown that seroconversion rates following quadrivalent vaccination in HIV-infected children aged 7–12 years and WLWH aged 13–27 years are comparable to those observed in HIV-negative controls, with vaccine-induced immune memory persisting for up to four years (30). However, due to the risk of suboptimal and short-lived immune responses in HIV-infected individuals, vaccination schedules should be appropriately adapted. While the CDC recommends a two-dose regimen for the general population, expert guidelines strongly advocate a three-dose schedule (0, 1–2, 6 months) for HIV-positive individuals. Furthermore, additional booster doses have been shown to substantially sustain antibody titers over time (31).

Since HIV infection accelerates the carcinogenic process of HPV, shortening the screening interval has become a consensus. However, developing countries still face significant challenges, including low screening coverage, severe stigmatization, and resource scarcity (32). Currently, global screening strategies are in a critical phase of transition from morphological to molecular biology-based approaches, but they also face a trade-off between specificity and accessibility (33). (1) High-sensitivity HPV DNA testing: WHO, ASCO, and NCCN recommend it as the preferred screening method (34). However, in WLWH, due to high persistent HPV infection rates and clearance rates heavily influenced by immune status (CD4 count, viral load), single HPV testing tends to lead to high false-positive rates, resulting in unnecessary colposcopy referrals and overtreatment. (2) Visual Inspection with Acetic Acid (VIA): As a practical alternative for low- and middle-income countries, VIA offers advantages of immediacy and low cost. However, its sensitivity is limited and its subjectivity makes it difficult to meet the needs for precise early diagnosis. (3) New biomarkers and self-sampling techniques: To address the specificity bottleneck, techniques like DNA methylation and p16/Ki67 dual staining are showing promising applications.

An overview of cervical cancer screening guidelines for WLWH is shown in **Table 7**. Notably, recommended ages for cervical cancer screening in HIV-infected women vary across institutions. While the general population is advised to begin screening at 25 years, U.S. federal guidelines (CDC/NIH) recommend that HIV-positive women start cytological screening at 21 to establish a baseline and monitor rapid disease progression. In contrast, the WHO suggests initiating high-sensitivity HPV screening at 25 in resource-limited settings, based on health economic considerations.

**Table 7:** Overview of cervical cancer screening guidelines for HIV-infected women.

Domain	U.S. federal/professional guidance (CDC/NIH/HIVMA) (35)	WHO global guidance (for LMIC settings) (36)	General-population reference (USPSTF/ACS) (37, 38)
Target population	Women living with HIV	Women living with HIV	Average-risk women (no immunosuppression/high-risk history)
Age to start screening	Start no later than age 21; commonly initiated at/soon after HIV diagnosis in clinical practice (follow the guideline algorithm)	Start at age 25 for routine screening	Start at 21 years (USPSTF); 25 years (ACS)
Strategy for ages 21–29	Cytology (Pap) only is emphasized; typically annual initially; if three consecutive normal results, interval may be extended per guideline pathway	HPV DNA testing as the primary test (programmatic approach)	Cytology every 3 years (USPSTF); ACS begins routine screening at 25 and prioritizes HPV-based approaches when feasible
Strategy for ages ≥30	Options include cytology or co-testing (cytology + hrHPV); generally more frequent early screening, with possible extension to every 3 years after adequate consecutive negative results per HIV-specific pathway	HPV DNA testing as the main strategy; screening interval every 3–5 years (context-dependent)	Ages 30–65: primary hrHPV every 5 years (preferred where available), or cytology every 3 years, or co-testing every 5 years
When to stop screening	Not recommended to stop at 65; typically lifelong screening is advised due to persistently elevated risk	No fixed upper age limit specified; continue based on individual health status and programmatic considerations	May stop at 65 if prior screening is adequate and recent results remain negative (per criteria)
Self-sampling	May be considered in selected contexts where appropriate (wording depends on the specific guideline section/algorithm)	Promoted to increase coverage in HPV DNA–based programs (screen–triage–treat logic)	Increasingly incorporated as an option in guideline updates for HPV-based screening to improve access (details per guideline text)

By 2025, with the FDA’s approval of several test kits, both the ACS and USPSTF have regarded HPV self-sampling as a key strategy to improve screening coverage (39, 40). However, given the high rate of HPV co-infection among WLWH, self-sampling alone may lead to over-referrals (40). Therefore, alongside promoting self-sampling, it is essential to implement comprehensive cytological triage or a “Screen-and-Treat” strategy to address the significant challenges of triage management.

### 4.3 Treatment challenges and innovations

Bibliometric analysis indicates that research on HIV-associated cervical cancer has shifted from early disease characterization to prevention-oriented and technology-informed investigation. As shown in the keyword co-occurrence and conceptual structure maps (**Figures 4A**, **7A**), the field is mainly organized around HIV, human papillomavirus (HPV), cervical cancer, cervical cancer screening, and cervical intraepithelial neoplasia, together with epidemiological terms such as prevalence and risk. Citation-burst analysis shows that early attention focused on acquired immunodeficiency syndrome, tumor virus infections, papillomavirus, and antiretroviral therapy, whereas recent hotspots include early cancer diagnosis, HPV vaccination, women living with HIV, cervical cancer screening, systematic review, and deep learning (**Figure 6C**). Overall, the field has moved toward screening, prevention, and more precise risk assessment in WLWH.

#### 4.3.1 The specificity of pre-cancerous lesions and surgical management

In WLWH, management of cervical precancer should follow risk-based principles while accounting for higher recurrence (41). ASCCP guidance for histologic HSIL (CIN2/3) generally prefers excision; ablation is acceptable only when the entire transformation zone and lesion are fully visualized, endocervical sampling is negative for CIN2+, and cancer is not suspected (42). If colposcopy is unsatisfactory, margins are unclear, or disease is persistent/recurrent, excision is recommended; hysterectomy is reserved for selected recurrent HSIL when repeat excision is not feasible or desired. For invasive cervical cancer, NCCN algorithms support standard stage-based radical surgery when appropriate, but perioperative risk rises with poor HIV control: viral load >30,000 copies/mL or CD4 <50/µL has been associated with ~3-fold higher postoperative complications (43). Preoperative ART optimization and intensified post-treatment surveillance are therefore critical (41).

#### 4.3.2 Challenges in chemoradiotherapy

For locally advanced cervical cancer, cisplatin-based concurrent chemoradiotherapy (CRT) remains the standard treatment regimen (44). Although WLWH generally exhibit initial treatment responses comparable to those of HIV-negative patients, their long-term outcomes are markedly poorer, with a 3.6-fold higher risk of recurrence. Beyond the elevated risk of disease progression, chemoradiotherapy (CRT) in WLWH is further complicated by reduced treatment tolerance, lower completion rates, and the additional challenge of coordinating antiretroviral therapy with anticancer regimens.

A major clinical concern is the increased incidence of severe treatment-related toxicity in WLWH. The high frequency of “hematological toxicity” identified in bibliometric analyses is consistent with clinical observations. Shrivastava et al. reported that WLWH receiving chemoradiotherapy for cervical cancer had a significantly higher risk of grade 3/4 hematologic and gastrointestinal toxicities (45). This heightened susceptibility is partly attributable to HIV-associated immune dysfunction and impaired bone marrow reserve, which compromise patients’ ability to tolerate intensive chemoradiotherapy, thereby exacerbating treatment-related toxicities and undermining adherence. Although studies indicate that HIV-positive patients on stable combination antiretroviral therapy (cART) with effective viral suppression can achieve progression-free survival (PFS) comparable to HIV-negative individuals, maintaining treatment completion remains a significant challenge in real-world practice (46). A study from South Africa found that the proportion of WLWH completing at least four cycles of standard chemoradiotherapy was significantly lower than that of HIV-negative patients (58.5% vs. 76.1%) (47). This reduced adherence is likely multifactorial, reflecting not only treatment-related toxicity, but also inadequate social support, co-infections, and poor nutritional status (48–50).

Bibliometric keyword and cluster analyses indicate that “antiretroviral therapy” has remained a continuous research focus, while the emphasis has gradually shifted from survival extension to the management of DDIs and cumulative toxic effects. All patients diagnosed with gynecologic malignancies who are infected with HIV and have not previously received treatment should initiate antiretroviral therapy (ART) (51, 52). Studies have shown that the use of cART in WLWH can significantly restore immune function and reduce the incidence of opportunistic infections, mortality, plasma viral RNA load, and AIDS-related complications (53–55). For HIV-positive women with cervical cancer, the optimal treatment strategy generally involves platinum-based chemotherapy administered concurrently with or without radiotherapy, together with antiretroviral agents such as stavudine, tenofovir, and lamivudine. However, beyond the general increase in toxicity, the concurrent use of antiretroviral therapy and anticancer treatment introduces an additional layer of complexity through drug-drug interactions (DDIs) and overlapping toxicities (53). This is largely due to metabolic competition between anticancer and antiretroviral drugs—particularly protease inhibitors and non-nucleoside reverse transcriptase inhibitors—both metabolized via the cytochrome P450 system. Such interactions can delay chemotherapeutic metabolism, leading to toxic accumulation, while compromising cART efficacy and increasing the risk of viral rebound. Concurrently, overlapping toxicities may further exacerbate treatment-related adverse effects (**Table 8**). To mitigate these risks, current clinical strategies increasingly favor cART regimens containing integrase inhibitors when high-risk drug–drug interactions are anticipated. Continuous monitoring of hepatic, renal, and hematologic parameters is essential to safely co-administer antiviral and anticancer therapies.

**Table 8:** Antiretroviral therapy and chemotherapy drug interactions and toxicities.

Drug class	Drug	Interaction/toxicity	Recommendations
NRTIs	Tenofovir	Nephrotoxicity	Exercise caution when concurrently administering with platinum-based chemotherapeutic agents.
	Abacavir-Lamivudine	Hypersensitivity reaction	Baseline HLA-B*5701 genotyping is essential to mitigate the risk of hypersensitivity reactions associated with abacavir therapy.
	Zidovudine	Nausea, anemia, and myelosuppression	Concurrent administration with chemotherapeutic agents may potentiate myelosuppression.
PIs	Ritonavir	Neutropenia, Infections	Implementing cautionary measures during the administration of combined cyclophosphamide, doxorubicin, and etoposide is essential, along with systematic hematologic and cardiotoxicity surveillance.
NNRTIs	Efavirenz	DDIs	DDIs and extended pharmacokinetic Half-Life.
	Etravirine	DDIs	Unforeseen pharmacokinetic and pharmacodynamic interactions involving immunosuppressive medications such as cyclosporine, tacrolimus, sirolimus, and mycophenolate mofetil.
INSTIs	Raltegravir	DDIs	Reduced likelihood of drug-drug interactions; necessitates twice-daily administration.
	Elvitegravir	DDIs	Requires pharmacological enhancement with cobicistat, a potent CYP3A4 enzymatic inhibitor, which may precipitate significant drug-drug interactions with concomitant pharmacotherapies.
	Dolutegravir	Creatinine level alterations	A favorable pharmacokinetic interaction profile. Exercise caution when administering nephrotoxic chemotherapeutic agents, given their potential to elevate serum creatinine levels and impair renal function.

NRTIs, Nucleoside Reverse Transcriptase Inhibitors; PIs, Protease Inhibitors; NNRTIs, Nonnucleoside Reverse Transcriptase Inhibitors; INSTIs, Integrase Strand Transfer Inhibitors; DDIs, Drug-Drug Interactions.

### 4.4 Immunotherapy and drug application

Bibliometric analysis of HIV-associated cervical cancer shows increasing attention to immune-related aspects. Clustering and timeline analyses highlight core topics such as HPV, cervical intraepithelial neoplasia, screening, and women living with HIV, while recent bursts (2023–2025) emphasize vaccine development, early diagnosis, and screening strategies (**Figures 4A, C, D**). The emergence of vaccine- and host-pathogen–related keywords indicates growing interest in immunological mechanisms for prevention and management. Overall, the field is shifting from descriptive epidemiology toward approaches that leverage immune insights for risk stratification, early intervention, and more precise clinical strategies.

#### 4.4.1 Immune checkpoint inhibitors

Immune checkpoint inhibitors have demonstrated significant clinical translational potential. At the molecular level, HIV-related cervical cancer often exhibits high tumor mutational burden (TMB) and high PD-L1 expression, providing a biological basis for targeted intervention with PD-1/PD-L1 inhibitors, such as Pembrolizumab (56). Although early clinical trials of immunotherapy often excluded HIV-infected individuals due to concerns about incomplete immune reconstitution (57), the emergence of “Real-world Data” as a burst term in recent literature marks the completion of the evidence chain. Existing data indicate that ICIs not only show manageable safety in WLWH, but also exhibit promising antitumor activity, with no significant rebound in HIV viral load observed, confirming their feasibility in immunocompromised hosts (58).

#### 4.4.2 HIV protease inhibitors

Repurposing existing antiretroviral agents has revealed new opportunities in cancer therapy. HIV protease inhibitors (HIV-PIs), central to highly active antiretroviral therapy (HAART), exert direct antitumor and anti-angiogenic effects in multiple preclinical solid tumor models, independent of HIV infection. In cervical cancer models, HIV PIs such as saquinavir, lopinavir, ritonavir, and nelfinavir demonstrate multiple antitumor effects: they inhibit proliferation, reduce invasion and clonogenicity, and induce apoptosis and cell cycle arrest. These agents also interfere with HPV oncoprotein activity, thereby suppressing tumor growth both *in vitro* and *in vivo*. Beyond these direct effects, HIV-PIs modulate the tumor microenvironment and angiogenic signaling, including downregulation of matrix metalloproteinase-9 (MMP-9), normalization of tumor vasculature, and restoration of p53-mediated apoptotic pathways disrupted by HPV E6/E7. Together, these multifaceted actions suggest that HIV-PIs could serve as adjunctive anticancer agents alongside chemotherapy or immunotherapy, providing both antiviral efficacy and antineoplastic benefits in HPV-associated malignancies (59–63).

### 4.5 Deep learning and digital pathology

This hotspot corresponds directly to the newly emerging “Deep Learning” cluster (cluster #8) in **Figure 4A** and the deep learning-related themes that independently emerged between 2023 and 2025 in **Figure 7**. This trend represents the cutting-edge translational application direction in this field.

Driven by the WHO’s global elimination strategy, AI-based auxiliary diagnostic technologies (such as automated visual evaluation, AVE) are becoming key solutions to the pathology resource shortage in low- and middle-income countries (64–66). However, existing general AI models are primarily trained on data from the general population, making it challenging to adapt to cervical changes caused by chronic inflammation or opportunistic infections, which are commonly associated with HIV infection, resulting in higher false-positive rates (67). Consequently, developing AI models tailored for WLWH, including transfer learning approaches, has become a research priority. Simultaneously, smartphone-integrated mobile health (mHealth) systems are transforming screening strategies in sub-Saharan Africa. Highly cited studies, such as Stelzle D (2021), demonstrate intervention predictions derived from big data. Despite these advances, training data bias remains a major challenge. Future efforts will focus on creating inclusive multimodal databases and developing prognostic models specific to HIV co-infection, bridging the gap from population-based screening to individualized precision prevention.

## 5 Limitations

This study conducted a structured, multi-dimensional bibliometric survey using the WoSCC and Scopus databases. While the dual-source complementary strategy enhanced the robustness of the results, reliance solely on commercial indexing databases inevitably excluded certain grey literature and non-English local journals. Given that high-burden regions for HIV and cervical cancer are primarily located in sub-Saharan Africa and Latin America, restricting the language to English may have led to the omission of regionally significant studies.

In addition to data coverage concerns, the country-level collaboration analysis in this study was based on the national affiliations of all listed authors, which provides a reasonable indication of each country’s participation in the field but does not readily distinguish between leading and supporting roles in collaborative research. Some countries may contribute primarily through sample provision or clinical data collection, and their publication counts do not necessarily reflect true intellectual leadership. Relatedly, the use of author affiliation-based statistics may overestimate the research output of high-income countries while underestimating the contributions of low- and middle-income countries, which serve as actual sources of clinical data. This may obscure the full picture of the complex “global North funding—global South implementation” collaboration. Given the current limitations of mainstream bibliometric databases in reliably extracting information on corresponding authors, co-corresponding authors, and actual lead contributions, we did not undertake a sensitivity analysis based on corresponding author affiliations. Future studies could incorporate corresponding author networks, contributorship statements, and multidimensional collaboration indicators to enable a more nuanced assessment of international research partnerships.

At the analytical level, the overall keyword analysis lacks pre-layering by research type, which could mask subtle differences in the evolution logic of various subfields. Additionally, the inherent time lag of citation metrics means that several emerging research topics are represented primarily by recently published literature, and the relatively short window for citation accumulation may introduce a temporal lag in burst detection and the identification of research hotspots. Accordingly, interpretations of the momentum and scholarly impact of these newer thematic directions should be made with due caution. Finally, quantitative visualization tools cannot replace the qualitative assessment of the methodological rigor of individual studies.

Despite these limitations, this study objectively constructs the knowledge map of the field, effectively revealing the trajectory of paradigm transformation, and provides a solid macro-level reference for future precision intervention research and global health policy development.

## 6 Conclusion

From a bibliometric perspective, this study provides a systematic overview of the global research landscape and its evolution in the field of HIV-associated cervical cancer from 1990 to 2025. Our findings reveal a well-established international collaborative network led predominantly by the United States, with growing contributions from China, South Africa, and other emerging research actors. High-impact journals have played a central role in facilitating interdisciplinary exchange and shaping clinical standards. The thematic focus of the field has undergone a notable shift: early work centered largely on pathogenic mechanisms, whereas more recent research increasingly encompasses public health interventions, screening optimization, and vaccine evaluation within an integrated prevention and control framework. At the mechanistic level, the synergistic oncogenic interaction between HIV and HPV, together with the regulation of the tumor immune microenvironment, has become a core area of inquiry. Concurrently, the clinical translation of emerging technologies — including immune checkpoint inhibitors and artificial intelligence-assisted diagnostics — is reshaping approaches to precision diagnosis and treatment of this disease.

## Data Availability

The raw data supporting the conclusions of this article will be made available by the authors, without undue reservation.

## Supplementary material

Supplementary Figure 1 Comparative overview of dataset characteristics across Web of Science, Scopus, and the combined dataset.

Supplementary Figure 2 Double map overlay analysis visualizing citation relationships between disciplines.

Supplementary Table 1 Literature retrieval strategies employed across databases.

Supplementary Table 2 Annual citation trends comparison across datasets.

Supplementary Table 3 Top 20 keywords and their frequencies.
