# Supplementary figures and images for: Global research trends and hotspots in human immunodeficiency virus-associated cervical cancer (1990–2025): a multi-database bibliometric analysis
Source: Front Immunol. 2026 Jun 10;17:1835957. doi: 10.3389/fimmu.2026.1835957 (PMC13290717; doi:10.3389/fimmu.2026.1835957)

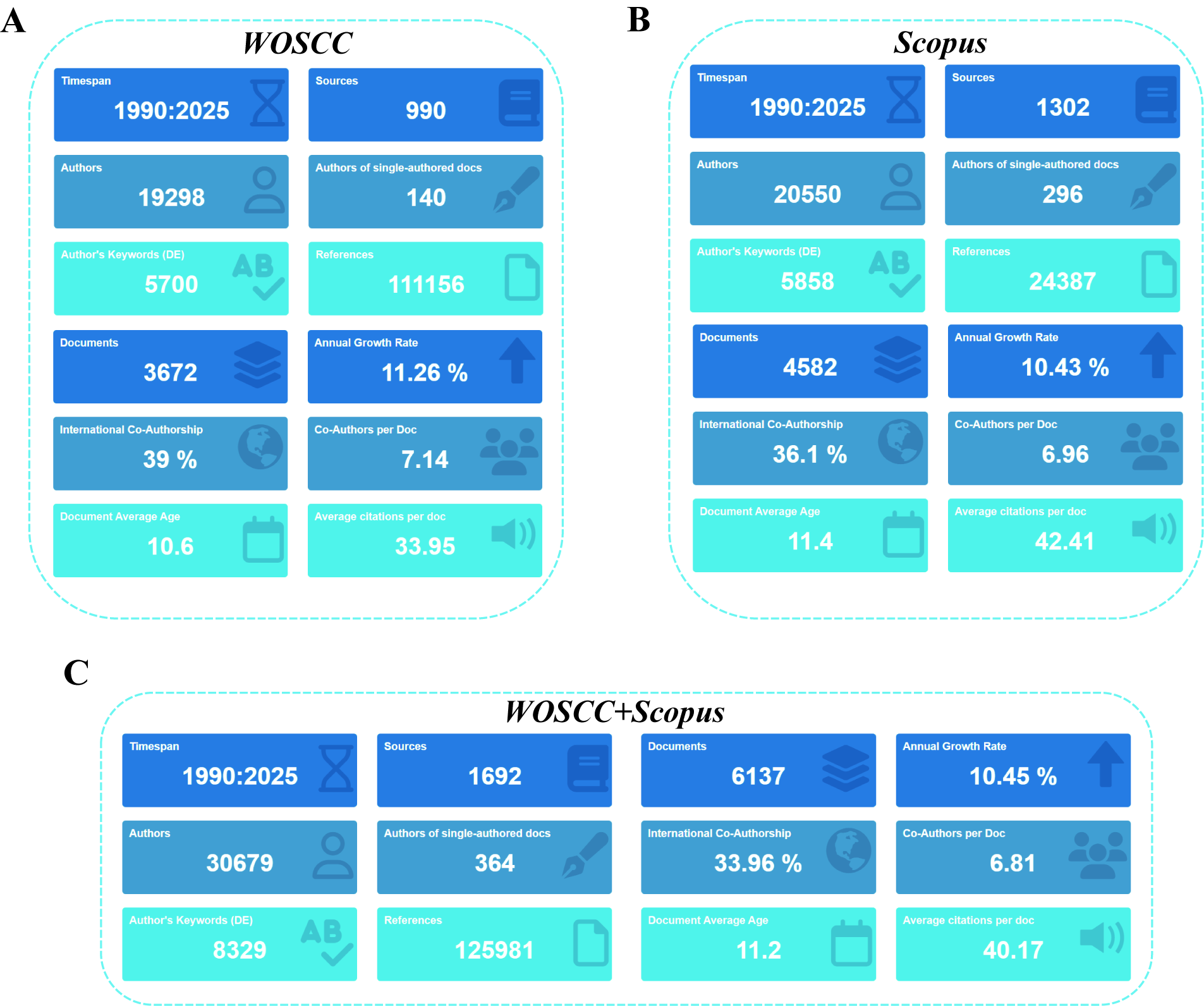

Supplement: Supplementary Figure 1 — Comparative overview of dataset characteristics across Web of Science, Scopus, and the combined dataset. [file Image1.tif]

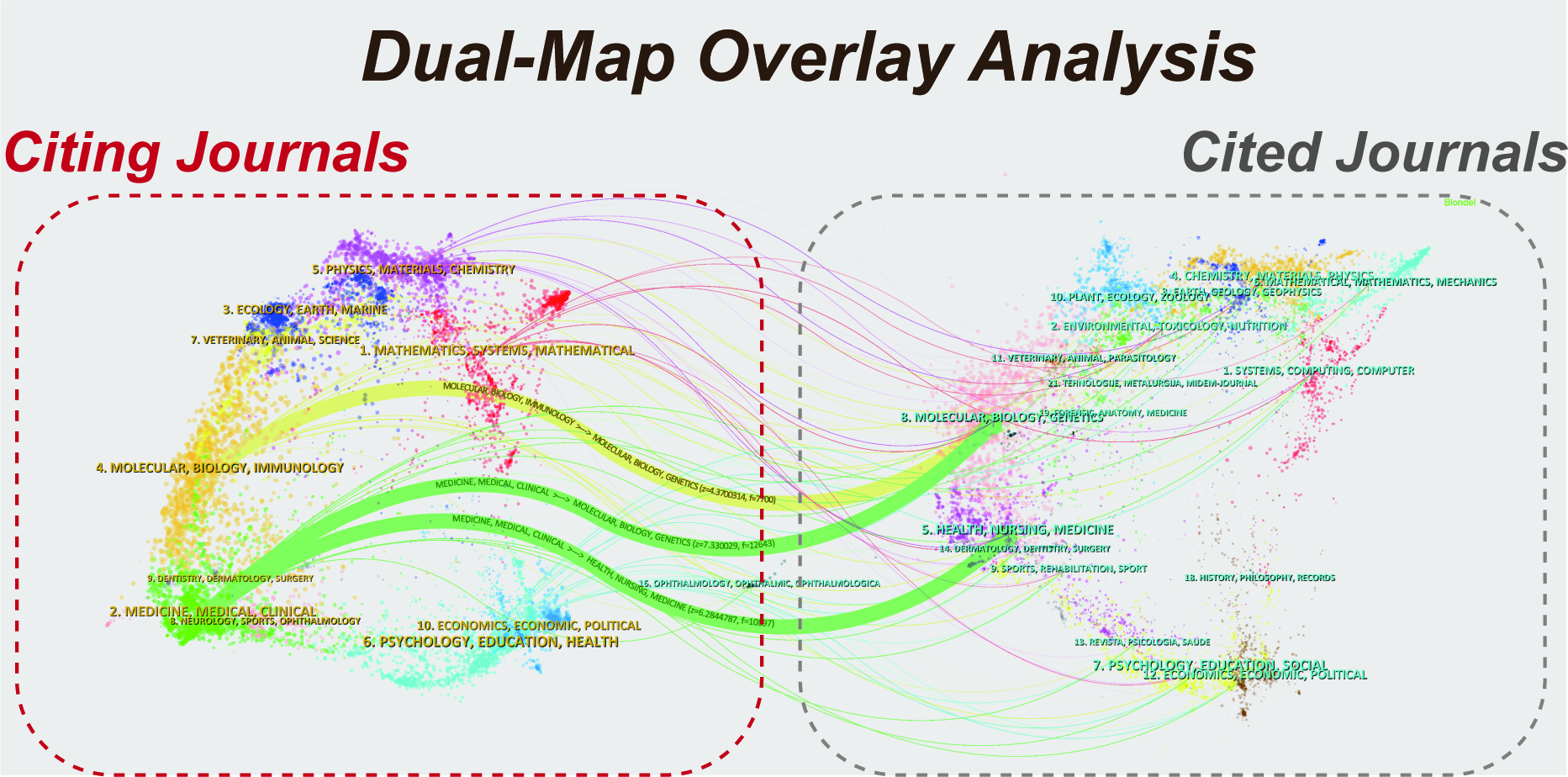

Supplement: Supplementary Figure 2 — Double map overlay analysis visualizing citation relationships between disciplines. [file Image2.tif]
