# Supplementary material for: Global research trends and hotspots in human immunodeficiency virus-associated cervical cancer (1990–2025): a multi-database bibliometric analysis
Source: Front Immunol. 2026 Jun 10;17:1835957. doi: 10.3389/fimmu.2026.1835957 (PMC13290717; doi:10.3389/fimmu.2026.1835957)
Supplement: Supplementary Table 1 — Literature retrieval strategies employed across databases. [file Table1.docx]

| **Database** | **Search Strategy** | **Search Date** | **Filtering Conditions** |
| --- | --- | --- | --- |
| WOSCC | TS1 = ("cervical cancer*" OR "cervix cancer*" OR "cervical carcinoma*" OR "cervical neoplasm*" OR "uterine cervical cancer*") ; TS2= (HIV OR "human immunodeficiency virus" OR AIDS OR "acquired immunodeficiency syndrome" OR "HIV-infected" OR "HIV-positive" OR "HIV-positive women" OR "HIV-infected women" OR "women living with HIV" OR WLWH); TS=TS1 AND TS2 | The literature search was conducted on January 28, 2026. | English-language publications from January 1, 1990, to December 31, 2025, including Articles and Review Articles. |
| Scopus | ( TITLE-ABS-KEY ( ( "cervical cancer*" OR "cervix cancer*" OR "cervical carcinoma*" OR "cervical neoplasm*" OR "uterine cervical cancer*" ) ) AND TITLE-ABS-KEY ( ( HIV OR "human immunodeficiency virus" OR AIDS OR "acquired immunodeficiency syndrome" OR "HIV-infected" OR "HIV-positive" OR "HIV-positive women" OR "HIV-infected women" OR "women living with HIV" OR WLWH ) ) ) | The literature search was conducted on January 28, 2026. | English-language publications from January 1, 1990, to December 31, 2025, including Articles and Review Articles. |
