# Supplementary material for: Global research trends and hotspots in human immunodeficiency virus-associated cervical cancer (1990–2025): a multi-database bibliometric analysis
Source: Front Immunol. 2026 Jun 10;17:1835957. doi: 10.3389/fimmu.2026.1835957 (PMC13290717; doi:10.3389/fimmu.2026.1835957)
Supplement: Supplementary Table 2 — Annual citation trends comparison across datasets. [file Table2.docx]

| **Year** | **WOSCC** | **Scopus** | **WOSCC+Scopus** |
| --- | --- | --- | --- |
| 1990 | 79.57 | 79.50 | 87.79 |
| 1991 | 33.20 | 23.36 | 26.07 |
| 1992 | 51.33 | 263.17 | 211.32 |
| 1993 | 54.45 | 54.90 | 50.40 |
| 1994 | 52.82 | 27.61 | 34.92 |
| 1995 | 33.12 | 21.52 | 25.55 |
| 1996 | 36.72 | 14.75 | 20.72 |
| 1997 | 87.67 | 59.53 | 62.04 |
| 1998 | 57.65 | 65.81 | 59.38 |
| 1999 | 50.47 | 48.14 | 49.84 |
| 2000 | 81.48 | 82.34 | 87.39 |
| 2001 | 74.88 | 68.49 | 70.63 |
| 2002 | 156.77 | 78.98 | 121.91 |
| 2003 | 43.13 | 53.90 | 47.79 |
| 2004 | 50.29 | 102.34 | 93.25 |
| 2005 | 65.26 | 60.96 | 56.85 |
| 2006 | 146.60 | 106.63 | 102.49 |
| 2007 | 48.34 | 48.40 | 49.82 |
| 2008 | 51.72 | 137.57 | 114.47 |
| 2009 | 62.77 | 68.55 | 61.09 |
| 2010 | 39.40 | 63.48 | 55.26 |
| 2011 | 45.72 | 49.93 | 50.59 |
| 2012 | 50.19 | 53.68 | 53.25 |
| 2013 | 37.12 | 41.76 | 41.80 |
| 2014 | 40.74 | 44.77 | 42.44 |
| 2015 | 30.88 | 81.21 | 69.93 |
| 2016 | 28.54 | 31.42 | 30.05 |
| 2017 | 34.54 | 72.67 | 62.98 |
| 2018 | 29.97 | 28.46 | 28.05 |
| 2019 | 27.54 | 33.69 | 35.01 |
| 2020 | 24.17 | 24.04 | 24.55 |
| 2021 | 20.65 | 22.34 | 21.85 |
| 2022 | 18.09 | 16.10 | 17.54 |
| 2023 | 11.87 | 12.70 | 13.31 |
| 2024 | 4.86 | 4.89 | 5.06 |
| 2025 | 1.17 | 1.07 | 1.11 |
