# Supplementary material for: Global research trends and hotspots in human immunodeficiency virus-associated cervical cancer (1990–2025): a multi-database bibliometric analysis
Source: Front Immunol. 2026 Jun 10;17:1835957. doi: 10.3389/fimmu.2026.1835957 (PMC13290717; doi:10.3389/fimmu.2026.1835957)
Supplement: Supplementary Table 3 — Top 20 keywords and their frequencies. [file Table3.docx]

| **Keywords** | **Count** |
| --- | --- |
| cervical cancer | 3230 |
| human immunodeficiency virus | 2514 |
| prevalence | 954 |
| papillomavirus infection | 952 |
| human papillomavirus | 875 |
| uterine cervical neoplasms | 824 |
| risk factor | 774 |
| priority journal | 763 |
| major clinical study | 735 |
| hiv infections | 674 |
| wart virus | 566 |
| controlled study | 543 |
| risk | 542 |
| uterine cervix tumor | 506 |
| cancer screening | 503 |
| cancer | 491 |
| cervical intraepithelial neoplasia | 472 |
| infection | 446 |
| breast cancer | 406 |
| antiretroviral therapy | 363 |
